# A randomised controlled trial of a psychoeducational group intervention for family and friends of young people with borderline personality disorder features

**DOI:** 10.1177/00048674231172108

**Published:** 2023-05-12

**Authors:** Jennifer K Betts, Mirra R Seigerman, Carol Hulbert, Ben McKechnie, Victoria K Rayner, Martina Jovev, Sue M Cotton, Louise K McCutcheon, Catharine McNab, Emma Burke, Andrew M Chanen

**Affiliations:** 1 Orygen, Melbourne, VIC, Australia; 2 Centre for Youth Mental Health, The University of Melbourne, Melbourne, VIC, Australia; 3 Melbourne School of Psychological Sciences, The University of Melbourne, Melbourne, VIC, Australia

**Keywords:** Borderline personality disorder, randomised controlled trial, carer, family, treatment

## Abstract

**Objective:**

Preliminary evidence indicates that interventions designed to support family and friends (‘carers’) of young people with early-stage borderline personality disorder effectively improve carer outcomes. None of these interventions have been tested in a randomised controlled trial.

**Method:**

This clustered, partially nested, randomised controlled trial was conducted at Orygen, Melbourne, Australia. Carers of young people (aged 15–25 years) with borderline personality disorder features were randomly assigned as a unit in a 1:1 ratio, balanced for young person’s sex and age, to receive a 15-day intervention comprising: (1) the three-session, in-person, Making Sense of BPD (MS-BPD) multi-family group programme, plus two self-directed online psychoeducational modules (MS-BPD + Online, *n* = 38), or (2) the two self-directed online psychoeducational modules alone (Online, *n* = 41). The primary outcome was ‘negative experiences of care’, measured with the Experience of Caregiving Inventory, at the 7-week endpoint.

**Results:**

A total of 79 carers were randomised (pool of 281, 197 excluded, 94 declined) and 73 carers (51 females [69.9%], M_age_ = 43.8 years [standard deviation, SD = 12.9], MS-BPD + Online *n* = 35 [47.9%], Online *n* = 38 [52.1%]) provided follow-up data and were included in the intent-to-treat analysis. The intent-to-treat (and per protocol) analyses did not find any significant differences between the groups on the primary (*d* = −0.32; 95% confidence interval = [−17.05, 3.97]) or secondary outcomes. Regardless of treatment group, caregivers improved significantly in their personality disorder knowledge.

**Conclusion:**

Delivering MS-BPD in conjunction with an online psychoeducational intervention was not found to provide additional benefit over and above access to an online intervention alone. In accordance with national guidelines, carer interventions should be routinely offered by youth mental health services as part of early intervention programmes for borderline personality disorder. Further research is warranted into which interventions work for whom, carers’ preferences for support and barriers to care.

## Background

Borderline personality disorder (BPD) is a severe mental disorder that usually has its onset during adolescence and early adulthood (Chanen and McCutcheon, 2013) and is associated with a range of poor short- and long-term outcomes (Álvarez-Tomás et al., 2019). This has led to the development of early intervention programmes for BPD that have demonstrated improved outcomes for young people (Chanen et al., 2020, 2022).

The families and friends (henceforth ‘carers’) of people with BPD features also report adversity in terms of elevated burden (or negative appraisal of the experiences of caregiving), distress, grief, disempowerment and mental ill health (Bailey and Grenyer, 2013, 2014; Kirtley et al., 2019; Scheirs and Bok, 2007). The burden reported by the carers of young people with BPD features is greater than that experienced by the carers of young adults with anorexia nervosa and carers of young people with first-episode psychosis (Cotton et al., 2022; Seigerman et al., 2020).

Recent systematic reviews have concluded that there is preliminary support for the effectiveness of interventions for carers of individuals with personality disorders, with evidence for reducing burden, emotional burnout, pain, grief, guilt, overload and distress, and for improving relationship skills and family climate (Guillén et al., 2021; Sutherland et al., 2020). However, the studies to date have been limited by non-randomised designs, lack of or inadequate comparison groups, small sample sizes, limited follow-up, and high treatment and research dropout rates. Indeed, only two carer interventions have been evaluated using a randomised controlled trial (RCT) design. ‘Staying Connected’(Grenyer et al., 2019) and Mentalisation-Based Therapy (MBT) Family and Carer Training and Support (MBT-FACTS) (Bateman and Fonagy, 2019) were each compared with a wait list control group, which have been criticised for inflating effect sizes (Patterson et al., 2016). Regardless, no treatment effect was found for burden. These RCTs evaluated interventions for carers of individuals with BPD of any age and any stage of disorder (Chanen et al., 2016; Hutsebaut et al., 2019); it is likely carers’ experiences differ as a function of these variables.

To the authors’ knowledge, only three published interventions have been designed specifically for the carers of young people and implemented within an early intervention framework: Making Sense of BPD (MS-BPD) (Pearce et al., 2017), Kindred (Gleeson et al., 2020) and MBT-Parent (Jørgensen et al., 2020). MS-BPD and Kindred are informed by cognitive analytic therapy (CAT) and a relational approach to BPD, while MBT-Parent utilises an MBT framework centred on attachment theory, emotion regulation and mentalisation. MS-BPD was tested in a repeated-measures pilot study, with 23 carers who attended the manualised, three-session, in-person, MS-BPD psychoeducational multi-family group programme. This programme was associated with a significant reduction in burden and with an increase in self-reported personality disorder knowledge (Pearce et al., 2017). Kindred was evaluated with a repeated-measures design with 15 carers who engaged with the 12-week online Kindred programme, which provides self-directed, asynchronous, psychoeducation modules; carer-to-carer (closed) social networking (e.g. a newsfeed, posts and comments); and input from specialist and peer moderators (e.g. a clinical psychologist and a trained family peer support worker with lived experience of caring for a young person with mental ill-health). Core themes include understanding BPD; communicating with their loved one; and supporting carer self-efficacy and well-being. Kindred was associated with a significant reduction in burden and emotional overinvolvement (Gleeson et al., 2020). MBT-Parent was investigated using an exploratory, retrospective study, nested within an RCT of adolescent treatment. Seventy-five carers were offered either six 90-minute MBT-Parent sessions over 12 months or treatment as usual (TAU) for carers, based on whether their young person with BPD features was randomised to MBT Group Therapy or TAU for the young person. The manualised, in-person, multi-family group MBT-Parent session content includes psychoeducation about BPD; role plays; and analysis of difficult young person-carer interpersonal events. No difference in burden or carers’ satisfaction with the young person’s treatment was identified between the carer groups, when assessed at a single time point, 3 months after end-of-treatment.

While these findings are promising, a rigorous evaluation of carer interventions using an RCT design with an active comparison group is warranted. Evaluations to date have relied on carers self-referring in response to public advertising (e.g. Grenyer et al., 2019) or arbitrary referrals initiated from within mental health services (e.g. Bateman and Fonagy, 2019; Gleeson et al., 2020; Pearce et al., 2017). As such, the true uptake of in-person carer interventions, and whether the interventions are acceptable to carers, is unknown and further investigation is required. In-person interventions require a greater commitment from carers (e.g. attendance at a set date and time, travel to the health service) and are resource-intensive for mental health services to deliver. A reliance on in-person carer interventions may be limiting the provision of, and engagement with, these programmes. Indeed, 62.3% of carers report wanting support but being unable to access it (Lawn and McMahon, 2015). Further exploration of the role of digital interventions as a means to address this unmet need is indicated.

This study aimed to evaluate the effectiveness of the in-person MS-BPD group, delivered in conjunction with an Online BPD psychoeducation programme (MS-BPD + Online), compared with the Online programme alone, in improving carer outcomes. It was hypothesised that MS-BPD + Online would be associated with superior results at the 7-week endpoint.

## Methods

### Study design

The study was a single-centre, parallel group, single-blinded, clustered, partially-nested RCT. Randomisation was by ‘unit’, that is, by the individual carer or group of carers per young person with BPD features. It was approved by the Melbourne Health Human Research Ethics Committee (HREC2014.105) and prospectively registered (ACTRN12616000304437). The trial protocol has been published (Betts et al., 2018).

### Study setting

The study was conducted at Orygen’s Helping Young People Early (HYPE) programme (Chanen et al., 2014) which provides specialist prevention and early intervention for young people (aged 15–25 years) with three or more *Diagnostic and Statistical Manual of Mental Disorders* (5th ed.; DSM-5) BPD criteria and who reside in western and northwestern Melbourne, Australia. HYPE offers general psychiatric care, clinical case management and individual CAT where indicated. HYPE uses non-judgemental language to actively engage carers and to provide psychoeducation about BPD and associated problems. Carers may be offered individual sessions with specialist family clinicians and/or family peer support workers. There are no specific exclusions for HYPE, although young people with first-episode psychosis usually receive treatment from Orygen’s early psychosis programme.

### Key inclusion and exclusion criteria

The young people with BPD features (‘clients’) were aged 15–25 years (inclusive) and the carers were their relatives, partners or friends. A carer’s participation was not dependent on their young person also consenting to participate.

### Discontinuation and withdrawal

Carers were discontinued or withdrawn if their participation interfered with appropriate clinical management of the client’s risk to self or others, consent was revoked, or an event (e.g. disruptive behaviour in the group setting) led to discontinuation from the intervention at the discretion of the investigators.

### Treatment conditions

The two treatment arms were Online and MS-BPD + Online and the treatment period was 15 days. Rather than utilise TAU or a waiting list as comparators, which may not be operationalised consistently or have the same effect within and between trials, this trial aimed for a more rigorous design by comparing two manualised, standardised, active interventions (Chanen, 2015). A digital intervention, Online, was selected as it might be more convenient for carers than an in-person intervention (thus facilitating access and engagement), and it is a less resource-intensive intervention for health services to implement (thus supporting its translation into routine clinical practice) (Betts et al., 2018; Gleeson et al., 2020).

Online comprised two self-directed modules of written material and video interviews with experienced clinicians, clients and parents (available at Orygen, n.d.). Module 1 (‘Introduction to Early Intervention for BPD’) content includes an explanatory model of BPD; difficulties associated with BPD in young people; and an introduction to early intervention for BPD. Module 2 (‘Caring For A Young Person with BPD – Information for Families and Friends’) includes normal development; causes of BPD; effects of BPD on young people’s support network; and supporting carer self-efficacy and well-being. Each module presents case studies and information about additional resources. The modules are accessible at all times of day, and they can be accessed multiple times and completed in stages. Treatment integrity was ensured by the Online module’s fixed design. Treatment completion for the Online group was defined as using the programme for at least 80% of the mean time it takes to complete the entire programme (i.e. 45.2/56.5 minutes).

MS-BPD + Online comprised the Online modules and MS-BPD, a manualised group programme developed for the carers of young people with early-stage BPD. Informed by the principles of CAT and the HYPE model of care, MS-BPD comprises three 2-hour in-person group sessions, run during the evening over three consecutive weeks. Session 1 (‘What is BPD?’) psychoeducation content includes an explanatory model of BPD; normal development; causes of BPD; the developmental context of BPD in young people; and the rationale for thinking about relationships in the treatment of BPD. Session 2 (‘BPD Treatment’) content includes an introduction to early intervention for BPD and common interpersonal difficulties and possible ways to resolve these. Session 3 (‘Looking After Yourself’) content includes managing risk from a relational perspective; supporting carer self-efficacy and well-being; and additional resources. Groups were facilitated by clinicians and (for Session 3) a lived experience expert, who has cared for a young person with mental illness. Treatment integrity was maintained via facilitator supervision, use of the manual and standardised programme resources (e.g. presentation slides). MS-BPD + Online treatment completion was defined as attending two or more MS-BPD sessions.

### Measures

The primary outcome was the combined negative appraisal subscales (sometimes referred to as ‘negative experience of caregiving’ or ‘burden’) of the Experience of Caregiving Inventory (ECI) (Szmukler et al., 1996). The secondary outcomes included combined ECI-positive appraisal subscales (‘positive experience of caregiving’) (Szmukler et al., 1996), coping (Endler and Parker, 1994), self-rated personality disorder knowledge (Davies et al., 2014), distress (Kessler et al., 2002), expressed emotion (Wiedemann et al., 2002) and quality of life (Endicott et al., 1993; Richardson et al., 2014). All measures demonstrated good to excellent internal consistency in this sample (Cronbach’s alpha 0.81–0.95).

### Procedure

Consecutive referrals to HYPE were considered for an invitation to participate in the RCT. Written informed consent was obtained from all participants (and from a parent/legal guardian for individuals under 18 years of age). Upon completion of baseline questionnaires, carers were randomly and consecutively assigned as a ‘unit’ (defined by their relationship to the young person with BPD features) in a 1:1 ratio, using randomised permuted blocking, and stratified by client’s sex and age (<18 years cut-point). The interventions were delivered in seven rounds, approximately 12 weeks apart, between March 2016 and September 2017. Follow-up questionnaires were issued to all carers at week 7, i.e., 4 weeks post-intervention (including those who had discontinued the intervention), and carers had up to 4 weeks to complete these. Data regarding the young person were extracted from their medical records.

### Statistical analysis

An intent-to-treat (ITT) analysis with all carers who provided follow-up data was conducted by a statistician who was blind to group allocation, followed by a per-protocol (PP) analysis with carers who completed treatment. A hierarchical linear modelling approach was used in order to account for the nested structure of the data (Lohr et al., 2014; Lohr and Schochet, 2014), with carers at level 1, unit at level 2 and intervention round at level 3, with the latter being applied only to those randomised to MS-BPD + Online. Difference scores were calculated for all outcome variables and used as dependent variables. Independent variables were group, unit and intervention round. Alpha was set at 0.05 and effect sizes were calculated by dividing the estimated impact statistic (an index of the estimated impact of the treatment intervention, compared with the comparison intervention, on the variable in question) by the standard deviation of the comparison group difference score (Lohr et al., 2014). Due to the complex, partially nested, clustered RCT design, Morris and DeShon’s (2002) approach for calculating within-subjects effect sizes, while correcting for dependence among means, was employed. This allowed for effect sizes of changes in scores from baseline to follow-up to be calculated by group. Paired sample *t*-tests were conducted to investigate the rate of pre- to post-intervention change by group.

The power analysis was based on assumptions that the average unit comprised 1.5 carers, the intraclass correlation coefficient within units was 0.2, the correlation within rounds was 0.03 and that each round would comprise 9.4 carers. Using these parameters, the maximum design effect was 1.24. Assuming a 0.5 correlation between baseline and outcome measures, a total sample of 54 units was deemed to have 80% power to detect a difference of 0.5 standard deviations (medium size difference) between the arms.

## Results

### Participant flow

**Figure 1.**
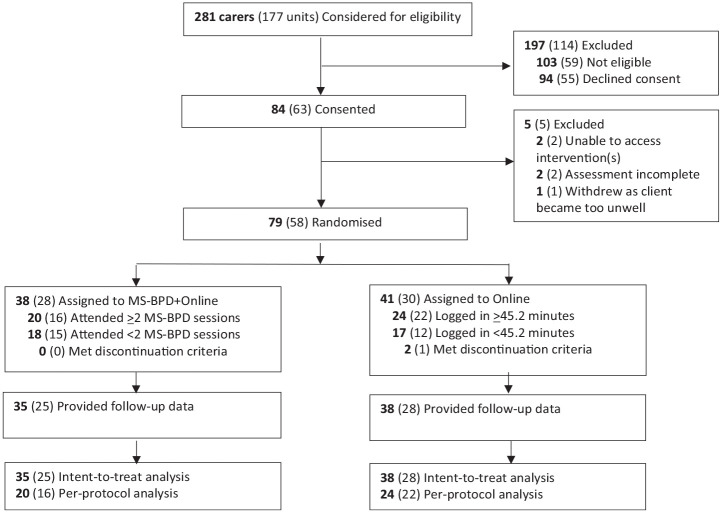
CONSORT diagram. Bold represents carers and () represents units. MS-BPD, Making Sense of Borderline Personality Disorder group program.

Two hundred and eighty-one carers were considered for inclusion in the study and 103 were excluded. The most common reasons for exclusion were that consent was not completed before the client was discharged from HYPE (*n* = 40), a member(s) of the carer unit had previously received the study intervention(s) (*n* = 28) and that the client was estranged from the carer and/or requested that the carer not be contacted (*n* = 14). Ninety-four carers declined to participate, most commonly citing work/study/family commitments (*n* = 39), access issues (travel distance, lack of a digital device and/or Internet connection [*n* = 12]) or their own poor physical and/or mental health (*n* = 12) as the barriers to participation in MS-BPD, Online or the study overall (Figure 1). A total of 58 units, comprising 79 carers, were randomised and 73 carers (92.4%) provided follow-up data. There were no significant differences between carers who did and did not provide follow-up data, χ^2^ (1) = 0.01, *p* = 0.923. Two carers (1 unit) randomised to Online reported that it would not meet their needs so discontinued and attended MS-BPD instead. Their data were included in the Online group in the ITT analysis and excluded from the PP analysis, as they did not meet the Online treatment completion criterion.

The 73 carers were family or friends of 53 clients. Data were not collected on seven clients because consent could not be obtained from them prior to discharge from HYPE (*n* = 4) or prior to the trial recruitment period closing (*n* = 2). One client declined to consent (*n* = 1). Thus, 46 (86.8%) clients consented to data collection.

### Participant characteristics

The carers were mostly middle-aged (M = 43.8 years [standard deviation, SD = 12.9]) females (69.9%) who were in an intimate relationship (61.6%; Table 1). Half of the carers (50.7%) were the clients’ mothers and the majority of carers were experiencing moderate to high socioeconomic disadvantage (64.4%), using a residential postcode-based index (Vinson et al., 2007). The majority of carers (38/65, 58.5%) received the intervention within 6 months of their young person first registering with Orygen.

**Table 1.** Baseline carer characteristics.

Variable	MS-BPD + Online (*n* = 35)	Online (*n* = 38)	Total (*n* = 73)
Age in years			
M (SD)	44.3 (12.9)	43.5 (13.1)	43.8 (12.9)
Range	20–58	15–72	15–72
Female, % (*n*)	71.4 (25)	68.4 (26)	69.9 (51)
Relationship with client, % (*n*)			
Mother	57.1 (20)	44.7 (17)	50.7 (37)
Father	14.3 (5)	18.4 (7)	16.4 (12)
Sibling	11.4 (4)	7.9 (3)	9.6 (7)
Non-related carer^a^	5.7 (2)	7.9 (3)	6.8 (5)
Partner	11.4 (4)	2.6 (1)	6.8 (5)
Friend	0 (0)	13.2 (5)	6.8 (5)
Grandparent	0 (0)	5.3 (2)	2.7 (2)
Highest level of education completed, % (*n*)			
Secondary or lower	51.4 (18)	39.5 (15)	45.2 (33)
University	34.3 (12)	47.4 (18)	41.1 (30)
Other	14.3 (5)	13.2 (5)	13.7 (10)
Employment, % (*n*)			
Employed	71.4 (25)	78.9 (30)	75.3 (55)
Unemployed	28.6 (10)	21.1 (8)	24.7 (18)
In a relationship, % (*n*)	62.9 (22)	60.5 (23)	61.6 (45)
Index of disadvantage, % (*n*)			
High	25.7 (9)	26.3 (10)	26.0 (19)
Moderate	45.7 (16)	31.6 (12)	38.4 (28)
Low	28.6 (10)	42.1 (16)	35.6 (26)

M: mean; SD: standard deviation; MS-BPD: Making Sense of BPD group intervention; BPD: borderline personality disorder.

a Includes step-parents, legal guardians and co-tenants.

The clients had a mean age of 18.8 years (SD = 3.1), were mostly female (71.7%) and half (50.0%) were diagnosed with BPD (Table 2).

**Table 2.** Client characteristics.

Variable	MS-BPD + Online (*n* = 22)	Online (*n* = 24)	Total (*n* = 46)
Age in years			
M (SD)	19.0 (3.1)	18.6 (3.0)	18.8 (3.0)
Range	15–25	15–24	15–25
Sex assigned at birth (female), *n* (%)	16 (72.7)	19 (79.2)	35 (76.1)
Gender identity, *n* (%)			
Male	6 (27.3)	5 (20.8)	11 (23.9)
Female	15 (68.2)	18 (75.0)	33 (71.7)
Gender diverse	1 (4.5)	1 (4.2)	2 (4.3)
BPD pathology			
Number of criteria met, M (SD)	4.7 (1.2)^a^	4.9 (1.4)^b^	4.8 (1.3)^c^
BPD diagnosis (⩾5 criteria), *n* (%)	8 (42.1)^a^	12 (57.1)^b^	20 (50.0)^c^
Additional diagnoses^d^			
Number of diagnoses, M (SD)	1.9 (0.9)	1.6 (0.9)	1.74 (0.9)
Any mood disorder, *n* (%)	17 (77.3)	16 (66.7)	33 (71.7)
Any anxiety disorder,^d^ *n* (%)	11 (50.0)	8 (33.3)	19 (41.3)
Any substance use disorder, *n* (%)	5 (22.7)	7 (29.2)	12 (26.1)
Posttraumatic stress disorder, *n* (%)	2 (9.1)	3 (12.5)	5 (10.9)
Antisocial personality disorder/conduct disorder, *n* (%)	2 (9.1)	1 (4.2)	3 (6.5)
Any eating disorder, *n* (%)	1 (4.5)	0 (0)	1 (2.2)
First-episode psychosis, *n* (%)	0 (0)	1 (4.2)	1 (2.2)
No additional diagnoses, *n* (%)	0 (0)	2 (8.3)	2 (4.3)

M: mean; SD: standard deviation; MS-BPD: Making Sense of BPD; BPD: borderline personality disorder.

a *n* =19.

b *n* = 21.

c *n* = 40.

d Excluding posttraumatic stress disorder.

### Treatment engagement

Of the carers in the ITT analysis randomised to MS-BPD + Online, 20 (57.1%) completed treatment, while 12 (34.3%) did not attend any MS-BPD sessions. The carers spent a mean of 48.9 minutes (SD = 146.9) logged into the online intervention, visiting a mean of 0.7 (SD = 1.7) times. Carers were not logged out after periods of inactivity. Six (17.1%) carers used the online intervention for more than 45.2 minutes and 25 (71.4%) did not log in at all. The carers had a mean of 0.9 (SD = 1.9) and 0.4 (SD = 1.1) phone contacts and 1.4 (SD = 1.9) and 0.5 (SD = 1.0) face-to-face contacts with the HYPE family work clinician and family peer support worker, respectively.

Of the carers in the ITT analysis randomised to Online, 17 (44.7%) completed treatment and 14 (36.8%) did not log in. Carers spent a mean total of 420.3 minutes (SD = 1594.6) logged into the online intervention, visiting a mean of 2.9 (SD = 3.8) times. They had a mean of 0.9 (SD = 1.9) and 0.3 (SD = 0.7) phone contacts and 0.6 (SD = 1.3) and 0.1 (SD = 0.4) face-to-face contacts with the HYPE family work clinician and family peer support worker, respectively.

### ITT analysis

Absolute repeated-measures effect sizes for pre-post differences by group ranged from very small (Cohen’s *d* = 0.08) to medium/large (*d* = 0.67) in MS-BPD + Online, and from no effect (*d* = 0.00) to a medium effect (*d* = 0.56) in Online (Sawilowsky, 2009) (Table 3). A significant pre-post rate of change was detected for personality disorder knowledge in both the MS-BPD + Online (*t* (34) = −3.98, *p* < 0.001) and Online (*t* (37) = −3.54, *p* = 0.001) groups.

**Table 3.** Pre-post differences on outcome variables by group.

Variable	Measure	Cohen’s *d* effect sizes		Paired sample *t*-test *p* value	
		MS-BPD + Online	Online	MS-BPD + Online	Online
		(*n* = 35)	(*n* = 38)	(*n* = 35)	(*n* = 38)
Negative experience of caregiving	ECI negative appraisal	−0.3	−0.04	0.09	0.81
Positive experience of caregiving	ECI positive appraisal	0.14	0.03	0.32	0.32
Task-oriented coping	CISS task scale	0.08	0.17	0.63	0.30
Emotion-oriented coping	CISS emotion scale	−0.2	0	0.25	0.64
Avoidance-oriented coping	CISS avoidance scale	−0.22	−0.08	0.19	0.43
Personality disorder knowledge	PDKASQ	0.67	0.56	<0.001	<0.01
Distress	K-10	−0.23	−0.02	0.19	0.90
Expressed emotion	FQ	−0.31	−0.06	0.08	0.63
Quality of life	AQoL-8D	0.11	0.01	0.28	0.79
Quality of life	Q-LES-Q-SF	0.15	0.01	0.59	0.80

MS-BPD: Making Sense of BPD; BPD: borderline personality disorder; ECI: Experience of Caregiving Inventory; CISS: Coping Inventory for Stressful Situations; PDKASQ: Personality Disorder Knowledge Attitudes and Skills Questionnaire; K-10: Kessler Psychological Distress Scale; FQ: Family Questionnaire; AQoL-8D: Assessment of Quality of Life–8 Dimensions; Q-LES-Q-SF: Quality of Life Enjoyment and Satisfaction Questionnaire–Short Form.

There were no significant differences detected between the MS-BPD + Online and Online groups for the primary and secondary outcomes (Table 4). Despite not reaching the level of statistical significance, MS-BPD + Online was associated with a small-to-medium effect size for burden (*d* = −0.32) and avoidance-oriented coping (*d* = −0.35), and a medium effect size for personality disorder knowledge (*d* = 0.46), compared with Online.

**Table 4.** Estimated impact of MS-BPD + Online on outcome measures.

	Baseline				Follow-up				Estimated intention-to-treat analysis				
	MS-BPD + Online (*n* = 35)		Online (*n* = 38)		MS-BPD + Online (*n* = 35)		Online (*n* = 38)						
	M	SD	M	SD	M	SD	M	SD	Estimated impact	95% CI	SE	*p*	Effect size
Negative experience of caregiving	104.1	33.6	102.0	33.5	96.8	37.8	101.3	36.6	−6.54	[−17.05, 3.97]	5.27	0.219	−0.32
Positive experience of caregiving	27.2	7.2	26.1	7.8	28.0	26.3	26.3	7.4	0.33	[−2.84, 3.50]	1.58	0.835	0.05
Task-oriented coping	3.1	0.5	3.1	0.7	3.2	0.5	3.2	0.6	−0.04	[−0.30, 0.22]	0.12	0.745	−0.09
Emotion-oriented coping	2.6	0.8	2.6	0.7	2.5	0.8	2.6	0.7	−0.05	[−0.57, 0.47]	0.20	0.816	−0.11
Avoidance-oriented coping	2.6	0.6	2.4	0.5	2.5	0.6	2.5	0.6	−0.18	[−0.42, 0.06]	0.12	0.141	−0.35
Personality disorder knowledge	2.8	0.8	3.1	0.8	3.5	0.7	3.5	0.6	0.29	[−0.24, 0.82]	0.22	0.231	0.46
Distress	23.5	8.4	25.3	8.0	21.9	9.3	25.1	7.0	−0.98	[−4.59, 2.62]	1.79	0.585	−0.14
Expressed emotion	2.7	0.6	2.6	0.5	2.6	0.6	2.5	0.5	−0.08	[−0.25, 0.08]	0.08	0.328	−0.23
Quality of life (AQoL-8D)	46.9	10.0	42.0	8.0	46.1	11.1	41.9	9.2	1.13	[−3.37, 5.63]	2.23	0.615	0.15
Quality of life (Q-LES-Q-SF)	68.6	12.02	65.9	11.37	70.4	14.1	66.0	12.7	−1.41	[−5.71, 2.88]	2.11	0.508	−0.19

MS-BPD: Making Sense of BPD; BPD: borderline personality disorder; M: unadjusted mean; SD: standard deviation; CI: confidence interval; SE: standard error; AQoL-8D: Assessment of Quality of Life–8 Dimensions; Q-LES-Q-SF: Quality of Life Enjoyment and Satisfaction–Short Form.

### PP analysis

No significant differences between the MS-BPD + Online and Online groups were detected for the primary and secondary outcome measures, when only treatment completers were analysed (all *p*s > 0.05).

## Discussion

The current study is the first RCT to evaluate early interventions designed specifically for carers of young people with BPD features and the first to use an active comparison intervention. It evaluated the effectiveness of the MS-BPD multi-family, group psychoeducation programme for carers of young people with BPD features by examining whether attending MS-BPD provided any benefit over and above access to an online psychoeducational intervention.

Two key findings arise from this study. First, both the MS-BPD + Online and the Online groups each experienced improvement. Second, no significant advantage was found for MS-BPD + Online, compared with Online, on the primary outcome of negative experiences of caregiving (aka burden) and on the secondary outcomes of positive experience of caregiving, coping, personality disorder knowledge, distress, expressed emotion and quality of life.

Both the MS-BPD + Online and Online interventions benefitted carers, despite an intervention period of only 15 days. Pre- to post-intervention effect sizes indicate change of very small to medium/large magnitude on all primary and secondary outcomes, except emotion-oriented coping, which appeared to improve in the MS-BPD + Online group but was unchanged in the Online group. Significance testing of the rate of change revealed that the secondary outcome of personality disorder knowledge improved significantly in both groups, but the rate of change for the primary and other secondary outcomes was not significant. Improvement in negative experiences of care, expressed emotion and personality disorder knowledge are consistent with pilot trials of both the MS-BPD and Kindred early intervention programmes (Gleeson et al., 2020; Pearce et al., 2017).

In conjunction with the findings from the Staying Connected and MBT-FACTS RCTs, this evidence suggests that negative experiences of care (or burden), knowledge about personality disorders and expressed emotion might be particularly responsive to treatment. These are also outcomes that the carers of people with personality disorder desire and value (Lamont and Dickens, 2021). Carer interventions should continue to target these areas, although greater understanding of the interplay between carer and client outcomes is needed, particularly with respect to expressed emotion, in which improvement might be advantageous to one group but possibly detrimental to another (Hooley and Hoffman, 1999).

Previous RCTs (Bateman and Fonagy, 2019; Grenyer et al., 2019) that have evaluated interventions for carers of individuals with BPD, of any age and at any illness stage, have also failed to detect any treatment effect for burden over and above any comparator, even when using a waitlist group, which is likely to inflate effect sizes. Yet, with the exception of MBT-Parent (Jørgensen et al., 2020), each of the carer interventions that have been formally evaluated have been associated with improvement on measures of burden (i.e. MS-BPD (Pearce et al., 2017), Kindred (Gleeson et al., 2020), MBT-FACTS (Bateman and Fonagy, 2019) and Staying Connected (Grenyer et al., 2019). The mechanism for such changes remains unclear. Given that carers often report insensitive, judgemental or even bigoted responses about BPD from health professionals (Acres et al., 2021; Chanen, 2021; Lamont and Dickens, 2021; Lawn and McMahon, 2015), it is possible that the experience of enrolling in a carer intervention (and study) might provide sufficient validation of the carers’ difficulties, instil hope and create an expectation of change, all of which might contribute to a reduction in burden (Bateman and Fonagy, 2019). Longitudinal data on the natural course of burden, from a cohort of carers who have not received an intervention, would help to elucidate this issue. It is also possible that successful treatment of the young person with BPD might lead to reduction in negative experiences of care. The two previous RCTs, which were not specifically directed towards the early intervention population, do not report detailed data on the treatment status of the family member or loved one with BPD. While the current study characterised the young person with BPD, no outcome data were collected. Given the consistency of the finding of reduced burden, future studies should consider measuring individual client outcomes as a possible mediator or moderator of reduction in negative experiences of care.

With regard to the secondary outcomes, evaluations of other BPD carer interventions have produced mixed results at treatment completion. Using a waitlist comparator, Staying Connected was associated with a faster reduction in expressed emotion, but not improvement in depression, anxiety or mental well-being (Grenyer et al., 2019). In contrast, MBT-FACTS was associated with an improvement in depression (rate only), anxiety (rate only) and mental well-being (both rate and extent of change) (Bateman and Fonagy, 2019). Taken together, these findings suggest that active carer interventions can reduce expressed emotion, but carers are likely to require individual treatment to adequately address their own mental health needs. Future studies should aim for longer follow-up periods to adequately assess such outcomes.

The current trial took place in a frontline clinical service, offering enrolment in the RCT to the family and friends of all young people attending the HYPE programme. Half of the carers declined the invitation to participate, often citing logistical issues (e.g. distance to travel, work/family commitments) as a barrier, suggesting the need for flexibility in the timing, location and nature of intervention for family and friends (Barr et al., 2020; Lamont and Dickens, 2021). While completion rates were satisfactory for an RCT in a frontline clinical setting, approximately one third of carers randomised to MS-BPD + Online did not attend any MS-BPD session. Similarly, approximately one third of carers randomised to Online did not log in to Online. The finding that a relatively simple, asynchronous and unmoderated online intervention was sufficient to improve outcomes for carers is encouraging, especially given the restrictions to clinical practice brought about by the COVID-19 pandemic. However, this is unlikely to suit or appeal to all carers and MS-BPD is likely to meet the needs of those who wish to share the experience of caring for a loved one with BPD, obtain mutual support and learn from the experiences of others. Further research is warranted into carer preferences and into other potential benefits of carer intervention that were not examined in the current study, such as hope, family empowerment, social connectedness, carers’ help-seeking behaviour for their own mental health needs, the carer-young person relationship, and the young person’s outcomes and service utilisation.

Strengths of this study include its prospective registration, treatment fidelity and excellent research retention rates. The trial had high ecological validity, as consecutive referrals to a frontline clinical service were invited to participate. Also, there were minimal exclusion and broad inclusion criteria, with young people of all genders included. Carers representing a range of relationships (beyond the mother–child relationship) were included, and the design enabled randomisation of multiple carers in a unit. The diagnosis of BPD in each young person was based on a gold-standard instrument, using a structured clinical interview. This was the first time that quality of life, coping styles and the positive experiences of caregiving were measured as outcomes in an RCT of BPD carer interventions. Inclusion of the positive experiences of caregiving was important, as the rewarding elements of caring for a young person with BPD are often overlooked (Seigerman et al., 2020). Evidence indicates that caring for a young person with BPD might not be uniquely associated with a global reduction in positive experiences. Rather, it might be associated with impairment only in specific domains, compared with caring for a young person with other severe mental illnesses, such as first-episode psychosis or anorexia nervosa (Cotton et al., 2022; Seigerman et al., 2020).

Study limitations include that Online was not programmed to have an automatic ‘timeout’ function after a period of inactivity (meaning that the duration of use per occasion is likely to be inflated), access to other carer resources/treatment during the trial was uncontrolled and only engagement with carer supports offered by Orygen was measured. The use of an active comparison condition, Online, avoided arbitrarily inflating the effect size associated with the experimental condition, MS-BPD + Online, but as the trial was only powered to detect medium-sized differences between the conditions, and any differences of a small magnitude might have gone undetected. The trial had a single, relatively short follow-up period and the outcome variables were exclusively self-report in nature. Future studies would benefit from the inclusion of objective measures, multi-method and/or multi-informant measures, and recruitment of larger samples that support the detection of differences of small effect size magnitude.

National clinical guidelines highlight the importance of supporting the carers of people with personality disorder (National Health and Medical Research Council, 2012; National Institute for Health and Care Excellence, 2009). In addition to MS-BPD, the current RCT adds online psychoeducation modules to the growing list of effective, evidence-based brief interventions that are designed for the carers of young people with BPD features, delivered within an early intervention framework. The online modules are a simple, scalable, ‘resource-light’ (i.e. free, publicly available, unmoderated) option that will likely meet the needs of some carers. Carer interventions should be offered routinely by youth mental health services as part of early intervention programmes for BPD. Further research into carers’ preferences for support and barriers to care is warranted, to select and deliver interventions that best meet carer needs.
